# A review of the studies on nonvisual lighting effects in the field of physiological anthropology

**DOI:** 10.1186/s40101-018-0190-x

**Published:** 2019-01-22

**Authors:** Tetsuo Katsuura, Soomin Lee

**Affiliations:** 1 Graduate School of Engineering, Chiba University, 1-33 Yayoi-cho, Inage-ku, Chiba, 263-8522 Japan; 2 Center for Environment, Health and Sciences, Chiba University, 6-2-1 Kashiwanoha, Kashiwa, Chiba, 277-0882 Japan

**Keywords:** Light, Nonvisual effect, Physiological anthropology, Color temperature, Light intensity, Monochromatic light, Intrinsically photosensitive retinal ganglion cell, Pupillary light, Melatonin suppression

## Abstract

Here, we review the history and the trends in the research on the nonvisual effect of light in the field of physiological anthropology. Research on the nonvisual effect of light in the field of physiological anthropology was pioneered by Sato and colleagues in the early 1990s. These authors found that the color temperature of light affected physiological functions in humans. The groundbreaking event with regard to the study of nonvisual effects of light was the discovery of the intrinsically photosensitive retinal ganglion cells in the mammalian retina in the early 2000s. The interest of the physiological anthropology scientific community in the nonvisual effects of light has been increasing since then. A total of 61 papers on nonvisual effects of light were published in the *Journal of Physiological Anthropology* (including its predecessor journals) until October 2018, 14 papers (1.4/year) in the decade from 1992 to 2001, 45 papers (2.8/year) in the 16 years between 2002 and 2017, and two papers in 2018 (January–October). The number of papers on this topic has been increasing in recent years. We categorized all papers according to light conditions, such as color temperature of light, light intensity, and monochromatic light. Among the 61 papers, 11 papers were related to color temperature, 20 papers were related to light intensity, 18 papers were related to monochromatic light, and 12 papers were classified as others. We provide an overview of these papers and mention future research prospects.

## Background

Light has a great influence on various organisms, including humans. Photoreceptors or photopigments, which accept light from the surrounding environment, are present not only in animals and plants, but also in bacteria and fungi, and are involved in several life-related processes. For example, phytochrome is a chromoprotein contained in plants, cyanobacteria, bacteria, and even in fungi, which accepts red light and far-red light [1]. In higher plants, phytochrome is involved in the regulation of flowering, germination, and shade avoidance reaction [2].

Rhodopsin is a vertebrate photopigment and is expressed in the rods. Opsins are photopigments that respond to red, green, blue, and violet and are present in the cones. Phylogenetically, red opsin (LWS) is the oldest photopigment followed by violet opsin (SWS1), blue opsin (SWS2), and green opsin (RH2). Finally, rhodopsin (RH1) was evolved from the green opsin [3]. During evolution, early mammals lost the green and blue opsins, which resulted in a loss of color vision in their nocturnal lifestyle. This is because, in nocturnal environments, color vision is not essential to survival, and it is more important to maximize the amount of light one can capture, rather than distinguish among spectral wavelengths [4]. However, in the diurnal primates, a mutation in the red opsin led to an alternate form of the green opsin [5]. Humans have violet (customarily called “blue”), green, and red opsins and rhodopsin. The human vision can perceive three colors (trichromacy) and is scotopic [6]. In addition to these visually related photopigments, an opsin (melanopsin, OPN4) is expressed in the mammalian intrinsically photosensitive retinal ganglion cells (ipRGCs) and is involved in various nonvisual or non-image-forming (NIF) responses, such as adjustment of circadian rhythm and pupillary constriction [7–9]. Melanopsin received its name because it was firstly identified in frog’s melanocyte [10]. Nevertheless, we know it now be closer to invertebrate’s rather than vertebrate’s photopigments [9, 11].

Physiological anthropology is one branch of anthropology and an academic discipline devoted to study the human biological nature from a physiological point of view. Ultimately, it aims to use this knowledge to improve people’s quality of life [12]. Research on physiological anthropology in Japan began with the study on the functions of the skeletal muscles and of the central nervous system in the 1950s. Since then, the research topics addressed have been expanding to several fields including human adaptability to the environment and human variability [12]. A large number of studies on environmental adaptability have been conducted in regard to the thermal environment—how has the human organism adapted to hot and cold environments? In the last 10–20 years, taking into consideration the developments on photopigments, there has been increasing interest around the light environment as well, especially in regard to nonvisual responses to light.

Herein, we outline the history and discuss the trends in the research on the nonvisual effect of light in the field of physiological anthropology. We mainly searched and reviewed the papers published in the *Journal of Physiological Anthropology* (JPA) and its predecessor journals (*The Annals of Physiological Anthropology*, *Applied Human Science*, *Journal of Physiological Anthropology and Applied Human Science*). We focused on JPA since it is the only journal on physiological anthropology in the world. We believe it may then constitute the most suitable source allowing to gather information about the current state-of-art information on this topic. However, it should be noted that the JPA does not include all studies from the perspective of the physiological anthropology field.

## The primordia of the research on nonvisual effects of light in the field of physiological anthropology

Research on the nonvisual effect of light in the field of physiological anthropology was begun by Sato et al. in the early 1990s; they mainly studied the influence of color temperature of light on human physiology, as measured by parameters such as the contingent negative variation (CNV) [13], blood pressure and critical flicker frequency [14], and heart rate variability (HRV) [15]. In 1941, Kruithof [16], a Dutch physicist, examined the influence of illuminance and color temperature on human pleasantness. Although he did not describe his methodological approach in detail, he might have not included any sort of physiological measurements. When Sato and colleagues began their research, the literature of architecture, illumination engineering, and ergonomics in Japan considered that the color temperature of the light should be no less than 4000 K, with desirable targets of more than 4500 K. Thus, a high color temperature of light was recommended. Sato wondered about this situation; at the same time, he had been asked to conduct research on this topic by a fluorescent light manufacturer. Therefore, he started conducting these studies to determine an optimal color temperature of light for humans. In these studies, Sato and colleagues found that CNV [13], diastolic blood pressure [14], and both Mayer-wave related sinus arrhythmia and respiratory sinus arrhythmia increased [15] in response to a high color temperature environment, when compared with a low color temperature environment. Bearing in mind that the work performance did not seem to improve under high color temperature, they clarified that the high color temperature lighting was not always desirable [13]. Supporting this notion, they found the level of activity of the sympathetic nervous system to be lower in a low color temperature environment [14], which made them to develop the idea that light with these characteristics may in fact be considered comforting light. These studies by Sato and colleagues pioneered the first observations in the laboratory experiments that differences in spectroscopic spectrum of light, which can be expressed by the color temperature, affect the physiological functions in humans. They greatly contributed to the subsequent development of the research on the nonvisual effects of light in the field of physiological anthropology.

Particularly groundbreaking in the field was the discovery of ipRGC in the mammalian retina in the early 2000s. The hypothesis of a third class of photoreceptor was raised in the 1990s and derived from the observation that retinal degenerate mice, which lost their cones and rods, still showed normal circadian responses to light [17]. The novel opsin, melanopsin, was identified in the retinal cells of *Xenopus laevis* (African clawed frog) [11] in 1998. The third class, non-cone, and non-rod photoreceptor in the retina of mammals was discovered by two research groups, Berson et al. [7] and Hatter et al. [18] in 2002. They found a small subset of retinal ganglion cells that were intrinsically photosensitive [7, 18]. Therefore, these cells were called intrinsically photosensitive retinal ganglion cells or ipRGCs [19, 20], which project to brain nuclei involved in nonvisual responses to light, such as the pupillary light reflex/response (PLR) and the circadian photoentrainment [21]. The photosensitivity of these cells depends on the presence of melanopsin [7, 18–22]. Hence, these cells are also called melanopsin-expressing retinal ganglion cells (mRGCs) [23].

**Fig. 1:**
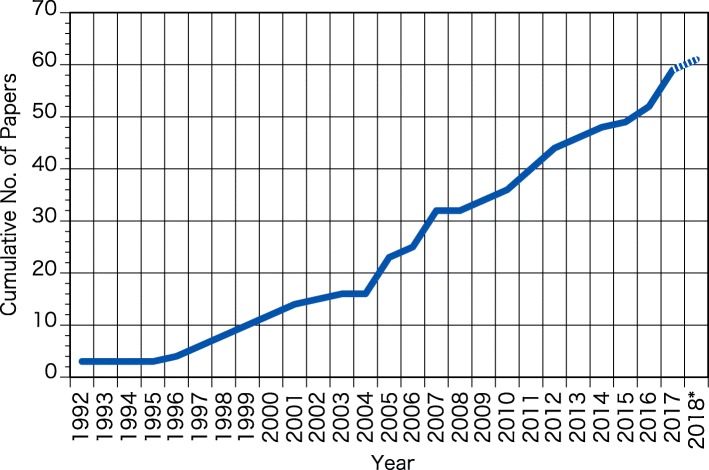
Cumulative number of papers on the nonvisual effects of light published in the JPA from 1992 to 2018* (asterisk denotes data from January to October were obtained in 2018)

**Fig. 2:**
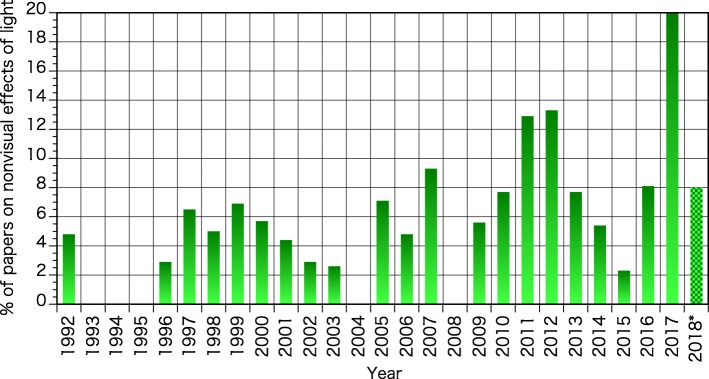
Proportion of papers on the nonvisual effects of light to the total number of papers published in the JPA from 1992 to 2018* (asterisk denotes data from January to October were obtained in 2018)

The discovery of ipRGC in the mammalian retina by Berson et al. [7] and Hatter et al. [18] had a strong impact on the study on nonvisual effects of light and was one of the main driving forces to increase research interested on the topic within the physiological anthropology community. Remarkably, a total of 61 papers on the nonvisual effects of light were published in the JPA (including its predecessor journals) by October 2018, 14 papers (1.4/year) in the decade from 1992 to 2001, 45 papers (2.8/year) in the 16 years from 2002 to 2017, and two papers in 2018 (January–October) (Fig. 1). This number has been increasing in recent years, which tacitly shows the increasing interest in this area of research. The proportion of papers on the nonvisual effects of light to the total number of papers published in JPA in this period are shown in Fig. 2. The percentages were varying between 0 to 6.9% of the total papers in each year and 3.6% on average in the first decade. Since 2002, the proportion has increased, with percentages varying between 0 to 20.0% yearly and an average of 6.7%.

**Fig. 3:**
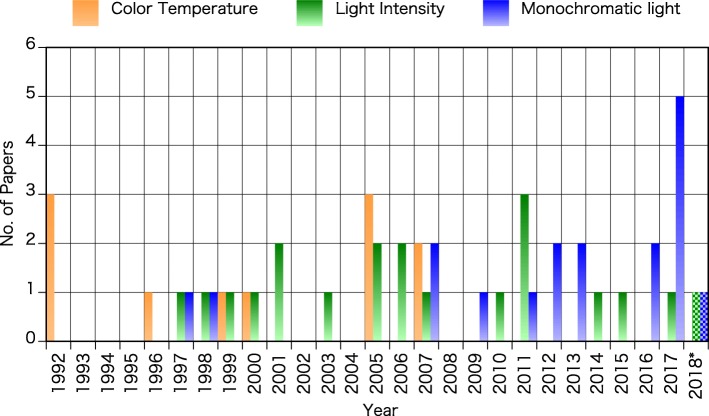
Number of papers for each lighting conditions published in the JPA from 1992 to 2018* (asterisk denotes data from January to October were obtained in 2018)

Herein, we classified each paper according to the light parameters explored in each manuscript, namely the color temperature of light, the light intensity, and the monochromatic light. We discuss in separate sections the evidence for each of these parameters. Those papers on both color temperature and light intensity were classified as color temperature, and those on both monochromatic light and light intensity, as monochromatic light. Field studies, laboratory experiments that did not use specified experimental light settings, and review articles widely discussing the nonvisual effects of light were classified as others. Figure 3 shows the distribution of the number of papers published in the JPA, from 1992 to 2018, according to the parameters of light inspected. Among all 61 papers, 11 papers were related to color temperature, 20 papers were related to light intensity, 18 papers were related to monochromatic light, and 12 papers were classified as others. As shown in Fig. 3, most papers on color temperature were published in the first half and in the middle of this period, with a more recent decline. Conversely, the number of papers examining the monochromatic light has been gradually increasing over time. After ipRGC discovery, monochromatic light has been considered an important topic of research. One to three papers on light intensity have been published yearly over a 15-year period.

We discuss below, in separate sections, the papers on color temperature, light intensity, monochromatic light, and others, as per the structure outlined above.

## Nonvisual effects of the color temperature of light

The influence of the color temperature of light on human physiology was the first aspect researched in the field of physiological anthropology and began, as previously mentioned, in the early 1990s [13–15]. Since then, this topic has been actively studied in the field of physiological anthropology (Table 1). Effects of color temperature of light exposed before sleeping on body temperature and melatonin secretion during sleep were examined. It was reported that exposure to high color temperature light before sleep tended to suppress the decrease in body temperature and the melatonin secretion levels during sleep [24]. Another study has focused on the effects of color temperature on sleep stage, as measured by polysomnographic recordings. This study found the amount of stage 4 sleep to be attenuated in the early phase of sleep [25]. High color temperature light was also found to attenuate the suppressions of heart rate observed during sleep and to increase the high-frequency (HF) components of HRV during sleep [26]. Altogether, these studies suggest that exposure to high color temperature light before sleep may be detrimental.

**Table 1:** Summary of the studies on the nonvisual effects of the color temperature of light in the field of physiological anthropology published in the JPA from 1992 to 2018*

Author	Year	Condition		Parameters Measured
		Lighting	Others	
Deguchi T, Sato M. [13]	1992	Color temperature: 3000, 5000, 7500 K		·CNV
		Illuminance: 1000 lx (fluorescent lamp)		·Reaction time
Kobayashi H, Sato M. [14]	1992	Color temperature: 3000, 5000, 7500 K	Addition task	·Blood pressure
				·CFF
		Illuminance: 320, 1000, 2000 lx (fluorescent lamp)		·Accommodation time of eye movement
Mukae H, Sato M. [15]	1992	Color temperature: 3000, 5000, 6700 K	Addition task	·Heart rate
		Illuminance: 100, 300, 900 lx (fluorescent lamp)		·HRV
Morita T, Tokura H. [24]	1996	Color temperature: 3000, 6500K		·Rectal temperature
		Illuminance: 1000 lx (fluorescent lamp)		·Melatonin
		Control: 50 lx (incandescent lamp)		
		Exposure time: 21:00-02:00		
Noguchi H, Sakaguchi T. [27]	1999	Color temperature: 3000, 5000 K		·HRV
		Illuminance: 30, 150 lx (fluorescent lamp) 22 min light exposure + 20 min sleep in darkness in the afternoon		·AAC
				·EEG
				·Subjective evaluation (drowsiness)
Yasukouchi A, Yasukouchi Y, Ishibashi K. [28]	2000	Color temperature: 3000, 5000, 7500 K	Experiment 1: Air Temp.: 28°C to 18°C during 30 min	·Rectal temperature
		Illuminance: 500 lx (fluorescent lamp)	Experiment 2: Air Temp.: 15°C for 90 min	·Skin temperature
				·Metabolic heat production
Yasukouchi A, Ishibashi K. [31]	2005	Review on nonvisual effects of color temperature		
Kozaki T, Kitamura S, Higashihara Y, Ishibashi K, Noguchi H, Yasukouchi A. [25]	2005	Color temperature: 3000, 5000, 6700 K	Sleep: 02:00-09:00 (<10 lx)	·Polysomnogram (EEG, EMG, EOG)
		Illuminance: 1000 lx (fluorescent lamp)		
		Exposure time: 19:30-02:00		
		Control: 2700 K (incandescent lamp)		
Katsuura T, Jin X, Baba Y, Shimomura Y, Iwanaga K. [29]	2005	Experiment 1:	Experiment 1:	Experiment 1:
		Color temperature: 3000, 7500 K	Taste stimuli (sweet, sour, bitter, salty)	·Taste threshold (sweet, sour, bitter, salty)
		Illuminance: 200, 1500 lx (fluorescent lamp)		·Saliva secretion
		Experiment 2:		Experiment 2: ·EEG
		Color temperature: 3000, 5000, 7000 K		
		illuminance: 1000 lx		
		Exposure time: 00:00-01:00 during night rest period		
Ishibashi K, Kitamura S, Kozaki T, Yasukouchi A. [26]	2007	Color temperature: 3000, 5000, 6700 K		·Heart rate
		Illuminance: 1000 lx (fluorescent lamp)		·HRV (FFT and CGSA)
		Exposure time: 19:30-02:00		
Jin X, Katsuura T, Iwanaga K, Shimomura Y, Inoue M. [30]	2007	Color temperature: 3000, 7500 K	Taste stimuli (sweet, sour, bitter, salty)	·Electrogastrogram (EGG)
		Illuminance: 200, 1500 lx (fluorescent lamp)		
		Exposure time: 14:25-ca.17:25		

*Data from January to October were obtained in 2018

One study has specifically evaluated physiological functions during short-term (22 min) exposure to light of different color temperatures and subsequently short-term (20 min) sleep after exposure [27]. It was found that the alpha attenuation coefficient (AAC) and mean power frequency of electroencephalogram (EEG) were lower during low color temperature light exposure, when compared with high color temperature. These findings suggest that low color temperature light is able to create a smooth lowering of central nervous system activity [27].

In respect to the influence of color temperature on body temperature regulation, it was found that lower color temperature environment enhanced the decrease in mean skin temperature when the air temperature was decreased from 28 to 18 °C and in a cold environment (15 °C). Evaluations of the rectal temperature confirmed this trend [28]. We have examined the influences of color temperature and illuminance on taste threshold and brain functions. Our results suggested that the sensitivity to sweet and bitter tastes increased under high illuminance and tended to increase under high color temperature light exposure. We also found the alpha1 band ratio in EEG to be increased and beta band ratio to be decreased under low color temperature during a nighttime break [29]. The influence of color temperature and illuminance on autonomic nervous system (ANS) activity during taste stimulation was also examined using electrogastrography (EGG). This study reported the ratios of the normal wave component of the EGG during sweet and salty taste stimulation to be significantly higher than those observed during bitter taste stimulation, with significant increases of this ratio under low color temperature exposure [30]. Yasukouchi and Ishibashi [31] reviewed the nonvisual effects of color temperature based mainly on their studies with special reference to sleep architecture, arousal level, and ANS (including parameters such as HRV, blood pressure, and body temperature regulation).

## Nonvisual effects of the light intensity

It has been well-known that the light intensity, which is expressed by illuminance, luminance, irradiance, etc., greatly affects the nonvisual responses [32]. This has also been a topic investigated in the field of physiological anthropology, with 20 papers published in the JPA over the years (Table 2). The vast majority of these papers inspected influences on the circadian rhythm [33–44]. A smaller number of papers have also addressed the influences of light intensity, when exposure occurs during a relatively short period, on the central nervous system (CNS) [45–47], the ANS as assessed by measurements such as HRV [46], PLR, nocturnal melatonin suppression [48], body temperature regulation [49], and serum tryptophan concentration and visuomotor and sensorimotor performance [50]. Two studies have specifically addressed effects on thermal regulation as measured by assessments of dressing behavior during cold exposure [51, 52].

**Table 2:** Summary of the studies on the nonvisual effects of the light intensity in the field of physiological anthropology published in the JPA from 1992 to 2018 (data from January to October were obtained in 2018)

Author	Year	Condition		Parameters measured
		Lighting	Others	
Higuchi S, Watanuki S, Yasukouchi A, Sato M. [45]	1997	Luminance: 10, 100, 320, 1000, 1800 cd/m^2^ Color temperature: 2800 K (tungsten lamp with diffuser and filters)		·CNV
				·EEG
Park SJ, Tokura H. [33]	1998	Illuminance: 200, 5000 lx (fluorescent lamp) Exposure time: 06:30–19:30		·Rectal temperature
Noguchi H, Sakaguchi T, Sato M. [46]	1999	Illuminance: 0.5, 1, 3, 10, 30 lx Color temperature: 3000 K (fluorescent lamp) Exposure time: 2 min	·Subjects: young, elderly ·Time slots of experiment 09:00–10:30, 10:30–12:00, 13:00–14:30, 14:30–16:00, 16:00–17:30	·HRV·EEG
				·Subjective evaluation (discomfort)
Kim HE, Tokura H. [51]	2000	Illuminance: 50, 3000 lx (fluorescent lamp) Exposure time: 09:30–14:30	Air temp.: 30 to 15 °C during 15:00–17:00 Elderly subjects	·Rectal temperature
				·Skin temperature
				·Blood pressure
				·Heart rate
				·Dressing behavior
				·Subjective evaluation (temperature sensation, thermal comfort)
Kanikowska D, Hirata Y, Hyun K, Tokura H. [34]	2001	Illuminance: 100, 3000 lx (fluorescent lamp) Exposure time: 07:00–18:00 first and second days: 100 lx, third day 3000 lx		·Rectal temperature
				·Melatonin
				·C-reactive protein
				·α1-antichymotrypsin
				·Transferrin
				·α2-macroglobulin
				·Haptoglobin
				·Ceruloplasmin
Wakamura T, Tokura H. [35]	2001	Illuminance: 3000 lx Color temperature: 5000 K (fluorescent lamp) Exposure time: 10:00–15:00 Ambient light: 50–300 lx	Subjects, 7 hospitalized elderly patients	·Melatonin
				·Bed time, get up time, time in bed, sleep start, sleep end, actual sleep time, immobile mins
Yokoi M, Aoki K, Shimomura Y, Iwanaga K, Katsuura T. [36]	2003	Illuminance: 120, 2800 lx (fluorescent lamp) Exposure time: 21:00–04:30 during sleep deprivation	Stroop color-word conflict test	·Melatonin
				·Rectal temperature
				·Skin temperature
				·EEG
				·Subjective sleepiness
Tokura H, Kim HE. [52]	2005	Illuminance: 10, 4000 lx Exposure time: 10:00–18:00 Illuminance: 10, 3000 lx Exposure time: 16:00–23:00	Air temp.: 30 to 15 °C during 20:30–22:30 Air temp.: 30 to 15 °C during 21:00–22:00	·Dressing behavior
				·Rectal temperature
				·Skin temperature
				·Melatonin
				·Subjective evaluation (temperature sensation, thermal comfort)
Higuchi S, Motohashi Y, Maeda T, Ishibashi K. [37]	2005	Illuminance: 1000 lx during 2 h from 2 h before peak melatonin concentration		·Melatonin
				·Questionnaire (Morningness-Eveningness Questionnaire)
				·Sleep diary (bedtime, rising time, sleep quality, etc.)
Yokoi M, Aoki K, Shimomura Y, Iwanaga K, Katsuura T. [38]	2006	Illuminance: 120, 2800 lx (fluorescent lamp) Exposure time: 21:00–04:30 during sleep deprivation	Stroop color-word conflict test	·Melatonin
				·Rectal temperature
				·Blood pressure
				·Heart rate
				·HRV
				·Task performance
Hyun KJ, Nishimura S, Tokura H. [39]	2006	Illuminance: 80, 5000 lx Second day: 07:00–23:00 in 80 lx Third day: 07:00–15:00 in 5000 lx, 15:00–23:00 in 80 lx		·Urine volume
				·Creatinine clearance
Yasukouchi A, Hazama T, Kozaki T. [48]	2007	PLR measurement: Illuminance: 1, 3, 30, 600 lx 5-min light exposure during 01:00–02:30 Melatonin measurement: Illuminance: 0, 30, 600 lx Exposure time: 01:00–02:30 Green (530 nm) (LED)		·PLR
				·Melatonin
				·Oral temperature
				·Blood pressure
Hirota N, Sone Y, Tokura H. [40]	2010	Illuminance: 2000 lx Exposure time: 07:00–15:00 Illuminance: 50, 2000 lx Exposure time: 15:00–24:00 (fluorescent lamp)		·Breath hydrogen production (unabsorbed dietary carbohydrates)
Kozaki T, Toda N, Noguchi H, Yasukouchi A. [41]	2011	Illuminance: 750, 1500, 3000, 6000, 12,000 lx Color temperature: 5000 K (fluorescent lamp) Exposure time: 09:00–12:00		·Melatonin (DLMO)
				·aMT6s concentration
Yoshinaga N, Fujita M, Tanaka YL, Nemoto S. [47]	2011	Illuminance: 0, 50, 200 lx Same, lowering and raising illuminance conditions Exposure time: 2 min + 2 min (fluorescent lamp)	Time zone of experiment: 11:00–17:00	·Somatosensory evoked potential (SEP)
				·Subjective evaluation (pain)
Kakitsuba N, Mekjavic IB, Katsuura T. [49]	2011	Illuminance: 1050 lx in winter 510 lx in summer and winter Color temperature: 5000 K (fluorescent lamp)	·Exercise: cycle ergometer (50% maximum work) ·Maintained mean skin temperature at 28 °C by perfusing a water-perfused suit with water at 25 °C.	·Core interthreshold zone (CIZ)
				·Rectal temperature
				·Skin temperature
				·Sweat rate
				·Oxygen uptake
Fukushige H, Fukuda Y, Tanaka M, Inami K, Wada K, Tsumura Y, Kondo M, Harada T, Wakamura T, Morita T. [42]	2014	Illuminance: > 5000 lx (Bright), < 50 lx (Dim) Exposure time: 07:30–18:00	Tryptophan intake: Rich 476 mg Poor 55 mg Poor*dim, Poor*bright, Rich*dim, Rich*bright	·Melatonin (DLMO)
				·Activity (sleep efficiency, sleep latency)
				·Subjective estimation (sleepiness, sleep maintenance, worries, integrated sleep feeling, sleep initiation)
Kozaki T, Kubokawa A, Taketomi R, Hatae K. [43]	2015	·Daytime Illuminance: < 10, 100, 300, 900, 2700 lx Exposure time: 09:00–12:00 ·Nighttime Illuminance: 300 lx Exposure time: 01:00–02:30 (fluorescent lamp) Color temperature: 4523 K		·Melatonin (DLMO)
Nagashima S, Yamashita M, Tojo C, Kondo M, Morita T, Wakamura T. [44]	2017	Illuminance: 50 lx (dim), 5000 lx (bright) Exposure time: 07:00–18:00 TRP*bright, placebo*bright, TRP*dim, and placebo*dim	Tryptophan (TRP) intake at breakfast	·Melatonin (DLMO)
Schobersberger W, Blank C, Hanser F, Griesmacher A, Canazei M, Leichtfried V. [50]	2018	Illuminance: 5000 lx, 6500 K (bright light) < 150 lx, 6500 K (office light) Exposure time: 30 min (07:40–08:10) (fluorescent lamp)	Sensorimotor and visuomotor performance tests	·6-sulfatoxymelatonin (aMT6s)
				·Tryptophan
				·Kynurenine
				·Reaction times

*LED* light-emitting diode

Tokura and colleagues found bright light exposure during daytime to enhance evening fall and morning rise of body temperature when compared to dim light exposure. The acrophase of body temperature change also occurred earlier under bright light exposure [33]. Interestingly, diurnal bright light exposure was also found to activate some acute phase proteins, to increase nocturnal melatonin secretion, and to accelerate the fall in rectal temperature observed during the first half period of night sleep [34]. Importantly, bright light was also found to increase the time spent in bed, actual sleep time, and melatonin secretion in hospitalized elderly patients [35]. Diurnal bright light exposure also increased urine volume and creatinine clearance [39], but evening exposure to bright or dim light after bright light exposure in the daytime had no varying effect on digestion or absorption of dietary carbohydrates in the following morning’s breakfast [40]. Kozaki et al. revealed the diurnal bright light exposure advanced the dim light melatonin onset [41]. Similarly, bright light exposure during the daytime was found to increased nocturnal melatonin secretion and to advance the phase of dim light melatonin onset [44]. Melatonin concentration during nocturnal light exposure after dim light exposure during daytime was decreased; however, exposure to bright light during the daytime blunted this response [43]. The combination of a tryptophan-rich breakfast and bright light exposure during daytime promoted melatonin secretion at night [42]. However, the intake of a tryptophan supplement during breakfast had no effect on nocturnal melatonin secretion [44].

Bright light exposure throughout nocturnal sleep deprivation suppressed the melatonin secretion and increase subjective ratings of sleepiness, and delayed the decline in heart rate and the increase in the theta and alpha wave activity [36, 38]. No significant correlation between individual differences in melatonin suppression by exposure to bright light during night and habitual bedtime in the 15 subjects in whom melatonin suppression occurred could be found [37]. Nevertheless, the habitual bedtime of the two subjects in whom melatonin suppression did not occur was earlier than that of the other subjects [37].

The following studies examined the influence of light intensity when exposure occurs during a relatively short period of time. The effects of luminance on CNV and spontaneous EEG during 10 min of simple reaction tasks were measured, and an inverted-U shape relationship between the changes in arousal level by light stimulation and resultant CNV was identified [45]. Noguchi et al. [46] studied the effects of exposure to different illuminances (0.5, 1, 3, 10, 30 lx) during 2 min after dark adaptation and found the LF/(LF + HF) of the HRV to follow a V-shaped trend, with a minimum on the 3-lx condition. The proportion of alpha wave activity on EEG fell markedly on 3 lx or higher illuminance conditions [46]. The effects of lowering or raising illuminance on somatosensory evoked potential (SEP) and subjective sensory evaluation were also examined. It was found that the SEP amplitude and the subjective sensory evaluation tended to decrease when illuminance was lowered and tended to increase when illuminance was raised [47]. The relationship between individual differences in nocturnal melatonin suppression induced by lighting and the individual differences on PLR were also investigated. Interestingly, it was found that subjects whose melatonin secretions were easily suppressed typically presented low PLR [48]. Kakitsuba et al. [49] examined the seasonal difference in the core interthreshold zone (CIZ)—defined as the range between core temperature at the onset of shivering and that at the onset of sweating—during winter and summer, in two different light exposure conditions. They found CIZ in summer to be higher than that observed during winter in both light exposure conditions. The effect of the morning bright light (07:40–08:10, 5000 lx) exposure on sensorimotor and visuomotor performance and serum tryptophan and its metabolites was evaluated [50]. These data suggest that morning bright light exposure has a limited effect on visuomotor and sensorimotor performance, but can potentially alter serum tryptophan concentration.

Tokura and colleagues [51] examined the effects of exposure to bright or dim light on dressing behavior and thermoregulatory responses in elderly people during exposure to cold in the afternoon (decreases from 30 to 15 °C). These authors found that the subjects tended to feel cooler and dressed more quickly with thicker clothes after dim light, when compared with bright light exposure. They also reviewed their own works about the effects of bright light exposure on dressing behavior in the cold and discussed the underlying physiological mechanisms [52].

## Nonvisual effects of monochromatic light

Studies on this specific topic have contributed with a significant number of papers (Table 3). The number of papers addressing this question has increased over the last years. Papers have mainly focused on the effects of monochromatic light on circadian rhythm [53–57] and pupillary constriction [58–63]. A few other papers have studied on HRV [64–66], time sense and respective event-related potentials [67], electroretinogram (ERG) [68], and body temperature regulation [69]. One paper has specifically focused on the influence of the colored paper [70].

**Table 3:** Summary of the studies on the nonvisual effects of monochromatic light in the field of physiological anthropology published in the JPA from 1992 to 2018 (data from January to October were obtained in 2018)

Author	Year	Condition		Parameters measured
		Lighting	Others	
Morita T, Tokura H, Wakamura T, Park SJ, Teramoto Y. [53]	1997	Blue (435 nm), green (545 nm), red (610 nm) Illuminance: 1000, 2500 lx Control 50 lx (incandescent lamp) Exposure time: 04:00–09:00		·Rectal temperature
				·Melatonin
Morita T, Tokura H. [54]	1998	Review on the influence of different wavelengths of light		·Rectal temperature ·Melatonin
Katsuura T, Yasuda T, Shimomura Y, Iwanaga K. [67]	2007	Red, blue Illuminance: 310 lx (fluorescent lamp)	Time zone of experiment: 10:00–18:00	·Time production (90 s, 180 s)
				·Event-related potential (P300)
				·Subjective evaluation (sleepiness, tension, fatigue, comfort, concentration)
Yoto A, Katsuura T, Iwanaga K, Shimomura Y. [70]	2007	Red, green, blue (sheets of paper in A2 size) Illuminance at surface of the color paper: 500 lx	Time zone of experiment: 10:00–17:00	·EEG
				·AAC
				·Blood pressure ·Subjective evaluation (lightness vs. heaviness, calm vs. excited, sleepy vs. not sleepy, cold vs. warm, etc.)
An M, Huang J, Shimomura Y, Katsuura T. [55]	2009	Blue (458 nm), green (550 nm) Irradiance: 9.8 μW/cm^2^ (halogen lamp with interference filter) Exposure time: daytime and nighttime (ca. 9.25 h before the respective averaged wake time) with 12 h out of phase each other		·Event-related potential (P300)
				·AAC
				·Subjective evaluation (sleepiness)
Higuchi S, Fukuda T, Kozaki T, Takahashi M, Miura N. [56]	2011	Light source: ceiling light (500 lx) Color temperature: 4200 K (fluorescent lamp) nonvisor cap (500 lx) red-visor cap (ca. 160 lx) blue-visor cap (ca. 160 lx) Exposure time: 23:00–03:00	Psychomotor vigilance task (PVT)	·Melatonin·Reaction time, no. of lapses (PVT)·Subjective evaluations (sleepiness, brightness)
Fukuda Y, Higuchi S, Yasukouchi A, Morita T. [68]	2012	468, 508, 593, 633 nm (LED) Masking-cone stimuli: 2000 ms (20 Hz) Test stimulus (mRGC or cone): 250 ms		·Electroretinogram (ERG)
Katsuura T, Ochiai Y, Senoo T, Lee S, Takahashi Y, Shimomura Y. [58]	2012	Blue (471, 467 nm), white (color temperature 3034 K) Irradiance: 12 μW/cm^2^ (LED)		·Pupillary constriction ·EEG ·Subjective evaluation (bluish, sleepiness)
Kakitsuba N, Mekjavic IB, Katsuura T. [69]	2013	Blue (436 nm), red (612 nm) Illuminance: 500, 1000 lx White (color temperature 5000 K) Illuminance: 1000 lx (fluorescent lamp)	·Exercise: cycle ergometer (50% maximum work) ·Maintained mean skin temperature at 28 °C by perfusing a water-perfused suit with water at 25 °C. ·Summer and winter ·Time zone of experiment 11:00–15:00	·Core interthreshold zone (CIZ) ·Rectal temperature ·Skin temperature ·Sweat rate ·Oxygen uptake
Lee SI, Hida A, Tsujimura S, Morita T, Mishima K, Higuchi S. [59]	2013	Blue (465 nm), green (536 nm), red (632 nm) Intensity: 12, 13, 14, 14.5 and 15 log photons/cm^2^ s (LED)	Time zone of experiment: 10:00–17:00	·Pupil size ·Melanopsin gene of single nucleotide polymorphism of rs1079610 (I394T):TT, TC, CC
Lee S, Ishibashi S, Shimomura Y, Katsuura T. [60]	2016	Blue (470 nm), green (532 nm) Irradiance: 10, 15, 20 μW/cm^2^ Pulse width: 1 ms (LED) Blue, green, blue + green	Time zone of experiment: 09:00–12:00, 13:00–16:00	·Pupillary constriction ·Subjective evaluation (sleepiness)
Yuda E, Ogasawara H, Yoshida Y, Hayano J. [64]	2016	Blue (10, 5, 2 lx) Green (71 lx) Red (39 lx) (OLED)	Time zone of experiment: 08:30–13:00	·Heart rate·HRV
Yuda E, Ogasawara H, Yoshida Y, Hayano J. [65]	2017	Blue (483 nm, 13 lx) Green (555 nm, 91 lx) White (158 lx) (OLED) Exposure time: 10 min + 5 min PVT under white fluorescent light (illuminance, 300 lx)	Psychomotor vigilance test (PVT) Time zone of experiment: 09:30–14:00	·Heart rate·HRV·Reaction time, minor lapse (PVT)
Dai Q, Uchiyama Y, Lee S, Shimomura Y, Katsuura T. [61]	2017	Blue (467 nm) Irradiance: 7.5, 15, 30 μW/cm^2^ Pulse width: 50, 100, 200 μs White (color temperature 2878 K, 14.75 μW/cm^2^) (LED) Exposure time: 12 min		·Pupillary constriction ·EEG ·Subjective evaluation (concentration, sleepiness, perception of blueness)
Yuda E, Ogasawara H, Yoshida Y, Hayano J. [66]	2017	Blue (485 nm, 8.02 μW/cm^2^) Orange (622 nm, 6.54 μW/cm^2^) (OLED) Exposure time: 30 min during lunch break 5 min of PVT under white fluorescent light (color temperature 4010 K, illuminance 450 lx)	Psychomotor vigilance test (PVT)	·Heart rate ·HRV ·Reaction time, minor lapse (PVT)
Lee S, Muto N, Shimomura Y, Katsuura T. [62]	2017	Blue (466 nm) Green (527 nm) Irradiance: 20 μW/cm^2^ Pulse width: 1 ms Inter-stimulus interval: 0, 250, 500, 750, 1000 ms		·Pupillary constriction
Lee S, Uchiyama Y, Shimomura Y, Katsuura T. [63]	2017	Blue (464 nm) Green (526 nm) Pulse width: 2.5 ms Photon density: 15.2 log photons/cm^2^/s Blue, green, blue + green		·Electroretinogram (ERG) ·EEG ·Visual evoked potential ·Pupillary constriction ·Subjective evaluations (bluish, greenish)
Kozaki T, Hidaka Y, Takakura JY, Kusano Y. [57]	2018	Blue (465 nm) Irradiance: 52 μW/cm^2^ 100-Hz flickering (10% duty tatio) non-flickering light (LED) Dim (< 3 lx) Exposure time: 01:00–02:30		·Melatonin

*LED* light-emitting diode, *OLED* organic light-emitting diode

We now discuss the studies on the effects of monochromatic light on circadian rhythm. Morita and Tokura et al. investigated the effects of exposure to blue, green, and red bright lights from 04:00 to 09:00 on the rectal temperature and melatonin secretion. They found that green light tend to promote an increase of rectal temperature and enhance the fall of melatonin secretion when compared to control dim and red lights [53]. The same authors also reviewed the papers on the influence of monochromatic light on human biological rhythms. In this review, the authors suggest that the effects of light on core temperature and melatonin secretion may vary depending on its wavelength, and hypothesize that the photoreceptors putatively responsible for transmitting light information that affects the biological rhythms are the M-cones [54]. In another study, authors examined the time-of-day-dependent effects of blue and green lights on human cognitive function. Blue light exposure caused a significantly larger P300 amplitude than that observed with green light, and the P300 amplitude at nighttime during blue light exposure was also larger than that occurred with the same color at daytime [55]. Higuchi et al. [56] studied the effects of a red-visor cap, which could cut the short wavelengths of light received from the upper visual field, on light-induced melatonin suppression, performance, and sleepiness during the night. They found the red-visor cap to be effective in preventing melatonin suppression, with no adverse effects on vigilance performance, brightness, or visibility. Recently, Kozaki et al. [57] evaluated light-induced melatonin suppression under dim, 100-Hz flickering and non-flickering blue light conditions at night (01:00–02:30). They found that melatonin concentration was significantly lower under non-flickering light than under dim light 15 min after the light exposure, whereas after 01:30, the mean melatonin concentrations were significantly lower under both 100-Hz flickering and non-flickering blue light than under dim light. These findings suggest that 100-Hz flickering light may have the same effect on melatonin secretion as non-flickering light.

The effects of monochromatic light on the pupillary constriction or PLR were evaluated in several studies. We have measured the pupil diameter, EEG responses, and the subjective bluish score during four lighting conditions, including a blue pulsed-light condition of 100-μs pulse width with a 10% duty ratio. Interestingly, we found that the pupillary constriction in the blue pulsed-light condition was greater than that observed in the steady blue light condition. Importantly, both conditions had identical melanopsin-stimulating irradiance [58]. We then examined the effects of separate and simultaneous exposure to extremely short pulses of blue and green lights at different irradiance levels on pupillary constriction. We found that pupillary constriction during the simultaneous exposure to blue and green lights was decreased when compared with separate exposure to blue light, despite the double irradiance intensity of the combination. These findings indicate that the effect of blue light on ipRGCs may be inhibited by simultaneous exposure to green light [60]. In another study, the effects of blue pulsed light with different combination of irradiance intensities and pulse widths in equal quantities (product of irradiance and pulse width) were tested for synergistic effects with white pulsed light. Pupillary constriction was found to be greater under high irradiance with short pulse width and under middle irradiance with middle pulse width, when compared with low irradiance with long pulse width condition. These findings indicate that higher intensity with shorter pulsed width of blue pulsed light produces a more significant influence on ipRGCs, even when the quantity of blue pulsed light is held constant [61]. The effects of both simultaneous and successive exposure to blue and/or green pulsed light on PLR were investigated using extremely short pulses (1 ms) of blue and green lights with inter-stimulus intervals (ISIs) ranging from 0 to 1000 ms. It was found that successive irradiation with pulses of blue and green lights at ISIs ≥ 500 ms induced pronounced pupillary constriction [62]. We also evaluated the effects of extremely short blue and green pulsed lights on visual evoked potential, pupillary constriction, ERG, and subjective evaluations. We found that pupillary constriction during the simultaneous exposure to blue and green pulsed lights was significantly lower than that observed during the blue pulsed light exposure, despite the double irradiance intensity of the combination. We also found the b/|a| wave of ERG during the simultaneous exposure to blue and green pulsed lights to be lower than that observed during the blue pulsed light exposure [63]. The association between melanopsin gene polymorphism (I394T) and pupillary light reflex under diverse photic conditions, including different intensities (12, 13, 14, 14.5, and 15 log photons/cm^2^/s) and wavelengths (465, 536 and 632 nm), was studied [59]. They found that the pupil sizes of the TC + CC genotypes were significantly smaller than those of the TT genotype under a blue (463 nm) light condition with 15 log photons/cm^2^/s, and the relative pupil constrictions of the TC + CC genotypes were greater than those of the TT genotype under both blue and green conditions with high intensities. These findings suggest that the melanopsin gene polymorphism (I394T) functionally interacts with pupillary light reflex, depending on light intensity and, particularly, wavelength [59].

The effects of monochromatic light on ANS, as assessed by HRV measurements, were studied in several works. Yuda et al. [64] recorded electrocardiogram activity during a 3-min darkness and a 6-min exposure to a blue, green, or red light. They found a greater decrease in HF power for blue light when compared to red and green lights. The decreases in HF power even lasted during the period of darkness that followed exposure. They analyzed HRV during blue, green and white light exposure and reported an increase in heart rate and a decrease in HF during blue light exposure, when compared with green and white lights [65]. In the same study, evaluating performance during a psychomotor vigilance test after exposure revealed a decrease in the number of minor lapses and in the variation of reaction times, after exposure to blue light when compared to the green light [65]. The same authors also compared the effects of blue and orange lights on HRV and behavioral alertness during the lunch break. They observed that blue light, when compared with orange light, enhanced autonomic arousal. This effect was not sustained and was not accompanied by changes in behavioral reports of alertness after exposure [66].

The effects of monochromatic light have been studied from other viewpoints as well. We examined effects of blue and red lights on time sense and correspondent event-related potentials and revealed that the 180-s time interval produced in the red light condition was shorter and the peak latency of P300 in the red light was also shorter than that in the blue light [67]. Fukuda et al. [68] compared the waveform of ERG by using the illumination system, which modulated stimulus levels to the ipRGCs and cones independently. They found that the response to the ipRGC stimulus was significantly higher than that observed to the cone stimulus, at approximately 80 ms after the onset of the stimuli, and tended also to be higher than that observed to the cone stimulus, at approximately 280 ms after the stimuli. CIZs during exposure to blue and red lights at 500 and 1000 lx in both summer and winter were compared in another study. The authors reported on a significant difference in the CIZs between red and blue lights at 1000 lx in the winter. Significant seasonal differences under red and blue lights were also observed at 1000 lx [69]. The effects of colored paper (blue, green, and red) on EEG, blood pressure, and subjective evaluations were investigated. It was found that AAC values were higher while looking at blue paper when compared to red. Alpha band power of EEG for red and green was also higher than that observed for blue [70].

## Other researches on nonvisual effects of light

In this section, we discuss the papers categorized as others, as previously described (Table 4). Tsumura et al. [71] compared the efficiency of carbohydrate absorption, as assessed by the breath hydrogen test, between Japan and Poland habitants. They found orocecal transit times for lactosucrose and the amount of undigested carbohydrate of minestrone of Japanese subjects to be significantly longer and larger, respectively, than those of Polish. The authors hypothesized that longer daytime and dimmer night light conditions in Poland could have affected morning digestive activity of Polish subjects, which presented enhanced absorption of dietary carbohydrate in the morning meal. Seasonal variations in melatonin secretion and surrounding light conditions were examined in another study. It was detected that peak values of melatonin secretion were higher in autumn than in other seasons. They also found that the relationship between peak level of melatonin secretion and the amount of daytime light exposure to more than 1000 lx was significant only in the autumn [72]. Nakade et al. [73] investigated the effects of tryptophan intake at breakfast and morning exposure to sunlight by using an integrated questionnaire in Japanese infants. They found that infants exposed to sunlight for 30–60 min in the morning showed distinctive shifting effects to morning type with protein intake, when compared to those exposed to sunlight for less than 20 min. The same authors also examined potential synergistic effects between tryptophan and vitamin B6 intake and morning exposure to sunlight in the same population. They found positive correlations between the Morningness-Eveningness (M-E) score and the amount of tryptophan intake and also between the M-E score and the vitamin B6 intake [74]. The positive correlation between M-E score and amount of tryptophan intake was only significant for infants who were exposed to sunlight for longer than 10 min after breakfast [74]. Melatonin secretion profiles and sleep patterns before and after cataract surgery were investigated in elderly patients. No significant differences could be identified in melatonin secretion, sleep parameters, or sleepiness before and after the surgery; however, the sleep efficiency of subjects with earlier wake-up and retiring times was higher than that observed in subjects with later wake-up and retiring times [75]. Lee et al. [76] analyzed the association between genotype of one polymorphism in the melanopsin gene (OPN4*Ile394Thr) and sleep/wake timing. They found that the sleep/wake timing of subjects with the CC genotype was significantly later than that of subjects with TT or TC genotypes. Adamsson et al. [77] examined the natural pattern of diurnal and seasonal light exposure and the seasonal variations in the circadian change of melatonin and cortisol concentrations in a group of Swedish office workers. They found large seasonal differences in the daily amount of light exposure across the year, alongside with a seasonal variation in melatonin concentration which presented a larger peak during the winter.

**Table 4:** Summary of other studies on the nonvisual effects of light in the field of physiological anthropology published in the JPA from 1992 to 2018 (data from January to October were obtained in 2018)

Author	Year	Condition		Parameters measured
		Lighting	Others	
Küller R. [78]	2002	Review on the influence of light on circadian rhythms and circannual rhythms		
Yasukouchi A. [79]	2005	Review on human adaptability to artificial light environment, based on evaluations from CNS, ANS, and biological rhythm.		
Tsumura Y, Hirota N, Tokura H, Sone Y, Lesinski F, Rutkowska D, Barinow-Wojewodzki A. [71]	2005		·Subjects: Japanese and Polish ·Tests were conducted at the summer of 2004 in Japan and Poland.	·Orocecal transit time (OOCT) for minestrone and for lactosucrose ·amount of undigested carbohydrate of minestrone (UCM)
Hanifin JP, Brainard GC. [80]	2007	Review on photoreception for circadian, neuroendocrine, and neurobehavioral regulation.		
Ueno-Towatari T, Norimatsu K, Blazejczyk K, Tokura H, Morita T. [72]	2007	Field study		·Melatonin (every 3 h from 10:00 on Thursday to 07:00 on Friday in four seasons) ·Light intensity (every 1 min for 5 day in four seasons)
Nakade M, Takeuchi H, Taniwaki N, Noji T, Harada T. [73]	2009		Subjects: infants (2–6 years)	·Integrated questionnaire (Morningness-Eveningness Questionnaire, sleep habits, breakfast habits, mental health, morning exposure to sunlight)
Tanaka M, Hosoe K, Hamada T, Morita T. [75]	2010		Subjects: elder males and females studied before and after their cataract surgery	·Melatonin·Amounts of light exposure ·Activity (sleep efficiency, sleep latency) ·Wake-up time, retiring time
Nakade M, Akimitsu O, Wada K, Krejci M, Noji T, Taniwaki N, Takeuchi H, Harada T. [74]	2012		Subjects: infants (2–6 years)	·Integrated questionnaire (Morningness-Eveningness Questionnaire, sleep habits, breakfast habits, mental health, morning exposure to sunlight)
Crowley SK, Youngstedt SD. [81]	2012	Review on light therapy for perinatal depression		
Lee SI, Hida A, Kitamura S, Mishima K, Higuchi S. [76]	2014			·Melanopsin gene of single nucleotide polymorphism of Ile394Thr:TT, TC, CC ·Morningness-Eveningness Questionnaire ·sleep habits (bedtime, wake time, sleep duration)
Daneault V, Dumont M, Masse E, Vandewalle G, Carrier J. [82]	2016	Review on neural pathways underlying effects of light on NIF functions, and discussed eye and cerebral mechanisms associated with aging		
Adamsson M, Laike T, Morita T. [77]	2017	Field study	South of Sweden (56° N) February 2008 to January 2009 3-day/month, beginning at 12:00 on Tuesday and ending at 12:00 on Friday	·Melatonin ·Cortisol ·Light exposure (illuminance) ·Spectral composition of the exposing irradiance

Küller [78] reviewed the influence of light on circadian and circannual rhythms in 2002. In this review, he discussed topics such as shift work, jet lag, and seasonal affective disorder and referred to the effectiveness of bright light exposure to alleviate these problems. Yasukouchi [79] discussed the physio-anthropological approach to evaluate human adaptability to artificial light environment in a review article published in 2005. He summarized the studies on the light environment in the field of physiological anthropology. Hanifin and Brainard [80] elaborated on the history of the study of action spectrum effects on circadian, neuroendocrine, and neurobehavioral responses. This study also included mentions to the discovery of melanopsin and ipRGCs and also discussed the use of light therapies for clinical and nonclinical applications. Crowley and Youngstedt [81] discussed the rationale behind the use of bright light therapy for perinatal depression and the available evidence supporting its efficacy. Future directions for this line of research were discussed as well. Daneault et al. [82] reviewed the neural pathways underlying the effects of light on NIF functions and discussed eye and cerebral mechanisms associated with aging which may affect NIF light sensitivity. They also reported on the results of investigations about pupillary constriction and cognitive brain sensitivity to light in the course of normal aging.

## Future prospects for the research on nonvisual effects of light in the field of physiological anthropology

ipRGCs and melanopsin were discovered in the mammalian retina in the early 2000s. Studies on the nonvisual effects of light have become more popular since then. New findings in the field of photobiology, genetics, and other related disciplines are expected to prompt further developments in the research on the nonvisual effects of light in the field of physiological anthropology. Recently, it has been suggested that ipRGCs are involved not only in nonvisual functions, but also in visual functions [83–86]. Intriguingly, a small population of M1 ipRGCs (one of the five ipRGC subtypes) has intraretinal axon collaterals that project toward the outer retina and transmit luminance signals retrogradely, influencing retinal light adaptation [86]. Of particular interest has been observations that cone-derived color signals may influence nonvisual responses to light, such as circadian entrainment [87, 88] and PLR [89–91]. Woelders et al. [91] have demonstrated in 2018 that M- and S-cones provide inhibitory input to the pupillary control system, whereas L-cones and melanopsin response present an excitatory role. These findings support a subadditive response to light, where the effects of blue light are reduced by green or polychromatic light exposure [60, 63, 92, 93].

One of the main goals of the research in physiological anthropology is to build a truly adapted artificial environment based on the biological characteristics of human beings. In the early 1990s, Sato et al. [13–15] explored and provided supporting experimental evidence in regard to the disadvantages of using high color temperature lights. These studies were the main driving force of all subsequent research on the nonvisual effects of light. The results of these studies may have informed, for example, the current choices for using low color temperature lights in living rooms and bedrooms, and suggested the appropriate timing for light exposure in order to achieve sleep improvements and a healthy life. Furthermore, these results can also be used to define the wavelength of illumination and the transparency characteristics of the eyeglasses needed to achieve enough adaptability for daily activities. Once further knowledge is obtained, the potential applications of this research in daily life may be enormous.

## Conclusion

Following a seminal study by Sato and colleagues on the effects of color temperature on human physiology in the early 1990s, research on the nonvisual effect of light in the field of physiological anthropology began and has increased over time. The discovery of ipRGC in the mammalian retina in the early 2000s has renewed the scientific interest in this topic, and many interesting findings have been obtained also in the field of physiological anthropology since then.

The influences of color temperature of light exposed before sleep were studied. Detrimental effects of high color temperature light before sleep were found. Many other significant effects of color temperature of light were also found in other aspects of physiological anthropology, such as body temperature regulation, taste threshold, brain functions, or electrogastrogram study. Many studies on the light intensity have been conducted also in the field of physiological anthropology. Works on light intensity have mostly examined effects on the circadian rhythm and on the CNS and ANS activities, PLR, nocturnal melatonin suppression, and body temperature regulation. Studies on the influence of monochromatic light have been numerous, especially in recent years. Their main focus was the circadian rhythm and the pupillary constriction. A few other studies have examined the effects of monochromatic light on HRV, ERG, time sense, event-related potentials, and body temperature regulation. Quite a few papers were categorized into others. Some of them are comparative studies of the efficiency of carbohydrate absorption between Japan and Poland. One study also explored the relation between seasonal variations in melatonin secretion and surrounding light conditions. A genetic association study evaluated effects of genotype groups for one polymorphism in the melanopsin gene on sleep/wake timing.

Recently, it has been suggested that ipRGC may be involved not only in nonvisual functions, but also in visual functions. Moreover, it has been shown that cone-derived color signals influence nonvisual responses. Prompted by these new findings, research on the nonvisual effects of light in the field of physiological anthropology is expected to develop. This knowledge will be paramount to better understand how current artificial environments may affect human beings. From there, informed strategies of intervention may be derived to reduce potential detrimental effects, while preserving the benefits of many of these modern daily life achievements.

## Abbreviations

AAC: Alpha attenuation coefficient
ANS: Autonomic nervous system
CIZ: Core interthreshold zone
CNS: Central nervous system
CNV: Contingent negative variation
EEG: Electroencephalogram
EGG: Electrogastrogram
ERG: Electroretinogram
HRV: Heart rate variability
ipRGC: Intrinsically photosensitive retinal ganglion cell
ISI: Inter-stimulus interval
JPA: Journal of Physiological Anthropology
M-E score: Morningness-Eveningness score
mRGC: Melanopsin-expressing retinal ganglion cell
NIF: Non-image-forming
PLR: Pupillary light reflex/response
